# Cultural evolution creates the statistical structure of language

**DOI:** 10.1038/s41598-024-56152-9

**Published:** 2024-03-04

**Authors:** Inbal Arnon, Simon Kirby

**Affiliations:** 1 Psychology Department, Hebrew University of Jerusalem, Jerusalem, Israel; 2 School of Philosophy, Psychology and Language Sciences, University of Edinburgh, Edinburgh, UK

**Keywords:** Human behaviour, Evolution of language, Cultural evolution

## Abstract

Human language is unique in its structure: language is made up of parts that can be recombined in a productive way. The parts are not given but have to be discovered by learners exposed to unsegmented wholes. Across languages, the frequency distribution of those parts follows a power law. Both statistical properties—having parts and having them follow a particular distribution—facilitate learning, yet their origin is still poorly understood. Where do the parts come from and why do they follow a particular frequency distribution? Here, we show how these two core properties emerge from the process of cultural evolution with whole-to-part learning. We use an experimental analog of cultural transmission in which participants copy sets of non-linguistic sequences produced by a previous participant: This design allows us to ask if parts will emerge purely under pressure for the system to be learnable, even without meanings to convey. We show that parts emerge from initially unsegmented sequences, that their distribution becomes closer to a power law over generations, and, importantly, that these properties make the sets of sequences more learnable. We argue that these two core statistical properties of language emerge culturally both as a cause and effect of greater learnability.

## Introduction

Two features of language provide the fundamental elements upon which linguistic structure is built, but their origins are surprisingly still poorly understood. First, language is segmented into smaller parts that are recombined sequentially: sounds are combined to form words and words are combined to form sentences. Second, the frequency distribution over words is highly skewed. While the languages of the world differ in many respects, they are consistently similar in having parts, and having those parts follow a particular frequency distribution^1^. In this paper we show, using an experimental analog of cultural evolution, that these features of language make it more learnable, and furthermore that they arise in the process of cultural transmission as a consequence of language repeatedly being learned by multiple generations of language users.

A fundamental challenge for an infant acquiring language is to discover what the relevant parts are. In spoken language, unlike in many written languages, word boundaries are not clearly marked. While there are multiple cues to word boundaries (phonotactics, stress patterns, statistical information, e.g.,^2^), none of the cues are fully reliable, and all have to be discovered by the infant. Discovering the correct units is a crucial first step for learning how to productively combine them. How do infants discover the relevant units without knowing what they are looking for?

One answer to this puzzle is that children are able to pick up on the statistical regularities that act as a cue for word boundaries in speech. One such cue, that has been extensively studied, is the transitional probabilities between syllables^3^. The idea is that children can rely on low-level statistics about which syllable is likely to follow another as a way to extract word boundaries. If language is made up of a repertoire of parts that recombine, then the transitional probabilities within these units will be higher than the transitional probabilities across unit boundaries. This is indeed what happens in language: the transitional probabilities of syllables that form a word are higher than those of syllables that cross word boundaries^4^. This reflects the fact that many different sounds can appear after a word, because it can be followed by many different words, while fewer sounds can follow the start of a word. Take the sequence pretty baby as an example, there are many different words that can appear after pretty (e.g., car, boy, hat, cat, and many more) but there are only few sounds that can appear after pre and result in a possible English sequence (premature, precise, and some more). This makes the transitional probability of syllables within a word (of the syllable ty given pre in our example) higher than that of syllables across word boundaries (ba given ty). A wealth of experimental evidence shows that infants, children and adults can track these transitional probabilities and use them to segment a novel continuous speech stream into its constituent parts (see^5^ for a review). The utility of transitional probabilities in discovering relevant units is not limited to language. Changes in transitional probabilities serve as a cue to unit boundary across domains: high transitional probabilities lead to grouping elements together while low transitional probabilities lead to separating them. For example, infants can use the transitional probabilities between visual shapes to detect recurring units in a continuous stream of shapes^6^.

This statistical learning literature offers a solution to how language learners might segment whole utterances into parts, given that those parts already exist. In other words, if language is already made up of recombinable parts then statistical learning can extract them. But we are left with a puzzle. How does language end up being constructed of parts whose combination makes such a learning strategy effective? In other words, where do the parts come from? One intuitive answer is that language is made up of parts because of the nature of the meanings we want to convey with language, which are themselves made up of parts. In other words, the structure of language arises from a pressure for us to use language expressively to communicate about a world that has structure^7^. However, here we test a stronger hypothesis: that parts will emerge purely under pressure for language to be learnable, even if there were no meanings for language to convey. This is an appealing hypothesis to test because infants begin to segment language prior to them learning what the parts mean^2,8^. Furthermore, when we look beyond language to other learned systems, like music, we see the presence of parts that can be recombined even without a mapping onto meaning.

A second striking fact about the statistical structure of language has to do with the frequency distribution of the parts. If we look at the lexicon of any language, it is immediately apparent that not all words occur with equal frequency. The frequency distribution of words is, in fact, highly skewed. A small number of words have a very high frequency and there is a long tail of low frequency words. Moreover, frequency doesn’t decrease in a linear fashion. In fact, the frequency distribution of words in a language tends to follow a power law distribution, such that a word’s frequency is inversely related to its rank. This recurring distribution was first highlighted by Zipf, and is often called a Zipfian distribution^1^. Words follow a Zipfian (or near-Zipfian,^9^) distribution across spoken and signed languages^9–12^, in child-directed speech^13^, and across different grammatical categories within language^9,14^. There is ongoing debate about the source of such distributions and whether they reflect foundational properties of human cognition and communication^9,15–17^.

Interestingly, Zipfian distributions seem to provide a facilitative environment for learning. Word segmentation in an artificial language is improved when learners are exposed to a skewed distribution compared to a uniform one (where all elements appear equally often,^18,19^). Moreover, participants are best able to segment sequences into their constituent parts when the distribution of these parts has a skew similar to that found in real languages (as reflected in entropy, ^19^). Skewed distributions have also been found to facilitate visual statistical learning (discovering recurring visual triplets in a continuous stream,^20^); cross-situational word learning^21^; and learning novel grammatical categories^22^ and constructions^23^. Speakers seem to have a cognitive preference for such distributions: uniform distributions of made-up nouns become skewed when a story is transmitted from one participant to another^24^. Though there are relatively few studies that explore the impact of skew on learning, the ones that do, find that skew can be beneficial for learning a range of linguistic and non-linguistic relations. These findings show that Zipfian distributions can facilitate learning, but they leave us with a second question: why does this facilitative distribution arise in language?

Both of these recurring, consistent properties of language—having parts and having them follow a particular frequency distribution—have learnability consequences: They help learners discover and learn the relevant linguistic units. In this paper, we aim to demonstrate that the fact that these statistical properties of language facilitate learning is also the reason that they exist in the first place. In other words, the statistical structure of language is both cause and effect of increased learnability. This seemingly circular argument follows from a view of language as arising from a process of cultural transmission where the product of one generation’s learning is the target for the next. In other words, the language that a child is trying to segment is the result of a similar segmentation process that their care-givers and others in their language community went through in the past. Similarly, the outcome of their learning will go on to provide linguistic data for a future generation of learners. This process of iterated learning has been shown to explain the origin of a wide range of structural properties of language such as compositionality^7^, duality of patterning^25^, regularity^25^, lexical semantics^26^, and even non-linguistic structure such as drumming patterns^27^ and verse templates^28^. In general, iterated learning over multiple generations tends to lead to languages that are optimised for the very learning biases that the individuals transmitting those languages have^29,30^.

We propose here that the process of iterated learning, combined with a particular learning strategy—one that starts with wholes before discovering parts—will give rise to these two statistical properties of language. While the textbook description of language acquisition often highlights part-to-whole learning, where smaller units are combined into larger ones (e.g., syllables to words, words to sentences), there is growing evidence that whole-to-part processes—where wholes are later analysed into parts—also play an important role in language learning^30,31^. In particular, such processes are important for learning linguistic structure. Because infants initially don’t know where word boundaries are, or even that words exist, their early building blocks will include a mix of single words and multiword sequences that are only later segmented into their parts (e.g., what’s-this). Learning from larger units that are then segmented can facilitate mastery of the arbitrary grammatical relations that hold between words (like those between gender marked articles and nouns in many languages^32^). Adults, because they already know what words are, and know the words of their first language, will rely less on multiword units, with detrimental consequences for learning grammatical relations. Indeed, there is experimental evidence that infants extract multiword units from early on^33,34^; that adults do so less^35,36^; and that starting with larger units can facilitate learning grammar^37–40^. This literature illustrates the presence of whole-to-part learning in real language acquisition, and its utility for learning linguistic structure.

Building on the different literatures on statistical learning, iterated learning, and whole-to-part learning, we predict that distributional properties of language can, and will emerge through the process of cultural transmission. The consequence of a whole-to-part learning strategy being iterated over generations of cultural transmission will be a gradual emergence of sub-parts that are recombined from initially holistic languages^41,42^. The emergence of parts should be reflected in the statistical properties of the language, with higher transitional probabilities within units compared to across unit boundaries. Furthermore, if the skewed distribution of these parts is both a cause and effect of increased learning, we expect to see the distribution becoming increasingly skewed over time, getting closer to the power-law typical of language.

To test this idea, we will turn to an experimental analog of the cultural evolution of language in which participants learn a “language” that was generated by a previous participant and have to reproduce it. Their output becomes the input for the next participant. In our version here, the transmitted behaviour is not linguistic, but instead consists of sets of colour sequences. Each sequence is analogous to an utterance, with the set being the “language” to be learned. Crucially, the initial set of sequences is not structured: all colour combinations are equally probable, meaning the sequences are not made up of recurring parts. We want to see whether the initial set will come to have, through transmission, the two statistical properties found in language: having parts and having them follow a particular frequency distribution. We draw on the statistical learning literature to look for parts in the sequences (see details below).

Importantly, the task is explicitly framed as copying wholes rather than finding recombinable parts, and is designed to make copying the whole difficult, creating a pressure on the systems to change to become more learnable. We use a non-linguistic task for two reasons. First, in this task, both the wholes (the sequences) and the parts (should they emerge) do not have meaning. This allows us to test the hypothesis that having statistically differentiated parts, and having them skewed in a particular way can both emerge, and facilitate learning, independently of meaning. Second, using non-linguistic stimuli, especially whose individual elements (and combinations) lack meaning, minimises the risk that participants will simply transfer knowledge of the statistical structure of their own language onto the behaviours that are being transmitted culturally. Any recombinable parts that do emerge will be the result of the system adapting through iterated learning to the implicit biases of the learners rather than being imposed explicitly by participants.

## Methods

We will reanalyse the results of an iterated sequence learning experiment^43^ to test our hypothesis that the statistical structure of language emerges from the process of cultural evolution through iterated learning by whole-to-part learners. In the original experiment, our aim was to test whether abstract sequences transmitted through iterated learning would, over generations, become easier to learn. However, we did not analyse the internal structure of those sequences nor was the experiment set up to explore that internal structure. In this sense, reanalysing this dataset provides a particularly strong test for our hypothesis, as it removes the typical “researcher degrees of freedom” when an experiment is designed with a particular hoped-for result in mind.

The experiment is modelled after the electronic “Simon game” toy. Participants are shown a sequence of lights flashing on a two-by-two grid of four colours (red, green, yellow, blue). They are then prompted to immediately recall that sequence of lights by pressing the relevant buttons on the grid and given feedback in the form of a score based on how close their response was to the target. Unlike in the original game, where players first see only one flash, then two, then three, and so on, in our experiment, participants see an entire sequence and have to reproduce it.

Participants are organised into transmission chains, where the responses of participant *n* in a chain are used as the sequences that participant *n* + 1 has to copy (with the order of sequences randomised between participants). Each participant sees a set of 60 sequences. The set is seen twice, in two blocks, with the sequences randomly ordered within each block (participants are not aware there are two blocks, they see all the sequences one after another, with no break between blocks or sequences). We record and analyse their responses to the second block. The first participant in each chain is given a set of random sequences to copy, with the constraints that each sequence consists of 12 lights, and each of the four colours is equally frequent within the sequence. The initial random set is different for each chain. The length of the initial sequences was set at 12 as to be not too easy and not too hard: we want the sequences to be hard enough to copy so that errors are made but not too hard so that none of the initial sequence can be reproduced. The copied sequences are then transmitted to the next participant in the chain. If participants produce responses that are shorter than length 6, then that target sequence is presented again to the participant at the end of the block.

We run each chain for 10 generations, so we have 660 sequences to analyse per chain (one random set of 60 sequences, and 10 sets of 60 sequences produced by the participants). We analyse the results of six chains, all run using the exact same design (four were initially reported in^43^), and two were reported in^44^). This yields a total of 3960 sequences organised by generation and chain (60 sequences X ten generations X 6 chains + the six initial random sets, containing 360 sequences). Note that the task is not presented as one of copying a set of behaviours, that is, participants are not instructed to attend to properties of the set as a whole. Any systematicity that emerges across the sequences is therefore a result of an implicit bias affecting the evolving sets of sequences. As mentioned above, the task is designed to be hard, especially at the start of the chains: we expect participants to make many errors in reproducing the sequences, particularly in early generations. We ask whether those errors lead to the emergence of more structured (and easier to learn) sets of sequences.

## Results

**Figure 1:**
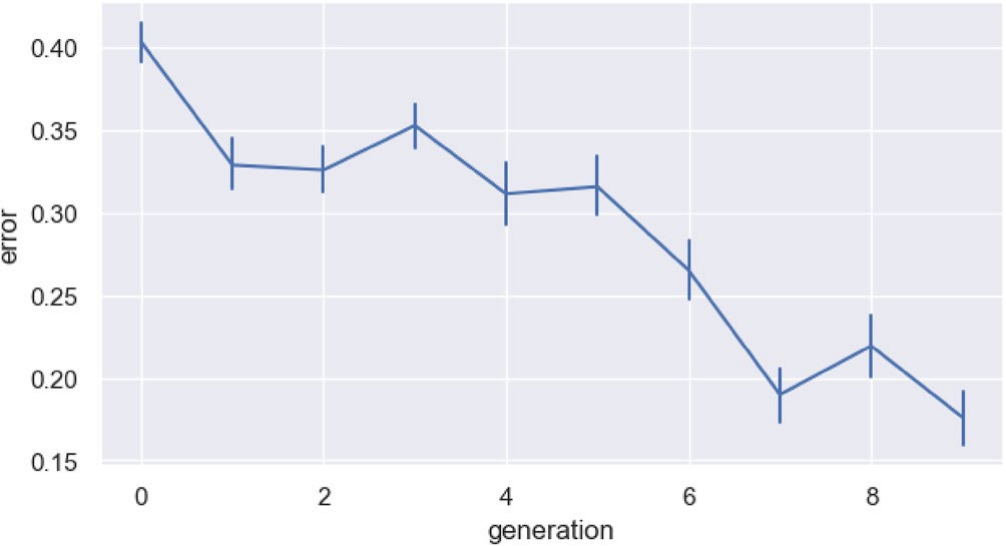
Change in error across generations in the experiment. The error value shown is an average across the different chains for each generation (error is calculated for each sequence, then averaged across all sequences in the set, then averaged across the six chains). Error bars are bootstrapped 95% CIs. There is a clear downward trend in error, indicating that the sets of sequences in later generations are easier to copy than those of earlier ones. The sequences have evolved to become more learnable.

**Figure 2:**
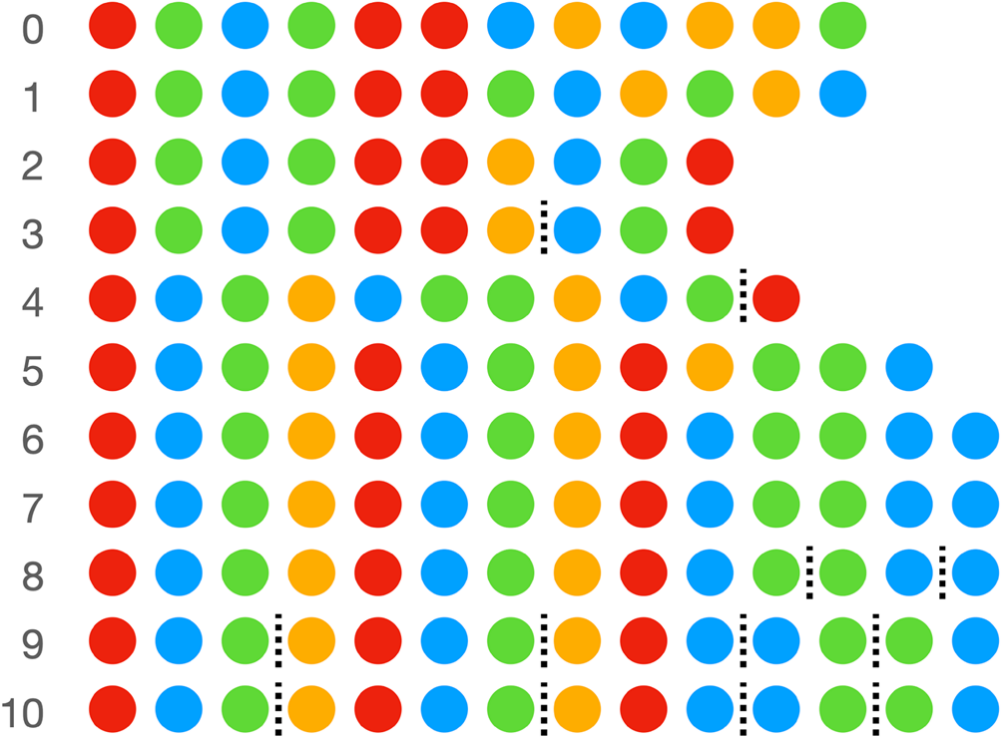
What happens to one particular sequence (out of 60) in one particular chain (out of 6) in the experiment. The numbers refer to generations in the experiment, with generation 0 indicating the initial random set, and subsequent generations being the sequences our participants produced. Initially, the random sequences are hard to copy and changes are introduced, but over generations the changes introduced make the sequences easier to repeat accurately. Dashed lines indicate particularly low transitional probabilities detected by our segmentation method described below, marking boundaries between emerging parts. The degree to which a single sequence like this is easy or hard to copy depends also on the other 59 sequences in the set. Equally, what counts as a segmentation boundary (low transitional probability) depends on the transitional probabilities calculated over the whole set.

The full dataset and code for recreating all the figures and statistical analyses is available at: https://github.com/smkirby/cultural_evolution_of_statistical_structure. Figure 1 shows that the error rate on the task decreases over generations. Error is calculated as the normalised Levenshtein distance between the target sequence and the sequence the participant produced, such that 1 would be a production with nothing in common with the target, and 0 would be perfect copying*.* We fit a linear mixed effects model predicting error, with fixed effect for generation, a random intercept for chain, random by-chain slopes for generation, and a random intercept for identity of original sequence in generation 0 (random by-chain slopes for sequence identity led to a singular fit, so this was simplified to intercept only). We find a significant reduction in error over generations (β = − 0.023, SD = 0.0032, *p* < 0.001). In other words, the sets of sequences are changing in such a way as to make copying those sequences easier for participants. This is a typical result for iterated learning experiments, where just by virtue of being transmitted from one generation to the next, learned behaviours change to become more learnable. Figure 2 shows an example sequence in one particular chain as it changes from one generation to the next (the figure also shows the parts discovered in each sequence using the segmentation method described below). Sequences that are easier to copy will tend to persist, whereas sequences that are harder to copy will change due to the introduction of errors. In this way, the sequences evolve to become more copyable over time.

### Segmentation emerges

We want to use this dataset of sequences to test whether parts emerge over generations. Recall that the statistical learning literature shows that changes in the transitional probabilities between syllables can be a cue to word boundary. Segmentation occurs when there is a drop in the transitional probabilities in a sequence of syllables. Such a drop is indicative of a word boundary since the transitional probabilities within words are on average higher than those between words. That is, when a language has recurring words, the transitions within words become more predictable than the transitions between words. We can ask if a similar pattern emerges in our data. In the Simon game task, the relevant elements are the four colours, so the transitional probabilities in question are the probability of a colour appearing given the context of previous colours. If parts are emerging, this should result in some colour transitions becoming more likely than others. Drawing on the statistical learning literature, we create a pipeline to detect dips in transitional probabilities in the sequence of colours that would reflect the emergence of parts.

One way to operationalise this is to look at the ratio between subsequent transitional probabilities as we scan across the elements in a sequence. In estimating the probabilities, we use the two previous elements: We estimate the probability of a colour given the two previous colours. Another approach would have been to use only the previous colour, but since there are only 4 colours, this would yield a very coarse estimate of the sequential structure of the data. Alternatively, we could have used the full conditional probabilities (i.e. all colours in the sequence up to this point). However, given the small data set size in each generation (only 60 sequences) this would have resulted in severe data sparsity problems. The use of two previous colours is therefore a compromise between these two constraints.

We calculate the maximum likelihood estimate of these transitional probabilities based on the full set of 60 sequences in a particular generation for a particular chain. So, for example, for the sequence “RYGR”, we estimate the probability of “G” given “RY” across the entire set. To do this we estimate the probability of occurrence of the trigram “RYG”, and divide it by the estimated probability of occurrence of the bigram “RY”. These probabilities are estimated using the entire set of 60 sequences. We then estimate the probability of “R” given “YG” in the same way. If we find that “G” is a highly likely continuation given “RY” but “R” is relatively unlikely given “YG”, then we have a cue to segment this sequence as “RYG | R”.

**Figure 3:**
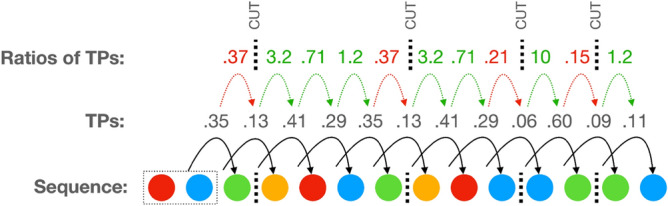
An example of our procedure for segmenting sequences. The example shows one of the sequences from the final generation of the experiment, along with inferred segmentation boundaries. Above the sequence we show the transitional probabilities for each colour given the previous two colours in the sequence. These transitional probabilities are estimated from the full set of 60 sequences for this particular generation in this particular chain (see text). We posit a unit boundary when there are drops in the transitional probabilities in the sequence. We operationalise this as a particularly low ratio between subsequent transitional probabilities. The ratios are shown in the top row of the figure. For example, the drop in transitional probability from 0.35 to 0.13 is lower than our threshold of 0.425 (see text) and is therefore indicative of a boundary between the first green and the first yellow in the sequence. Transitional probability ratios lower than our cutting threshold are shown in red, and ones above our threshold are shown in green. Note that a limitation of our method is that we can never place a boundary prior to the third colour in a sequence (because the transitional probabilities are based on the previous pair of elements, and the ratios on pairs of subsequent transitional probabilities).

We need some way of judging when a transitional probability is low enough to merit a segmentation boundary. One way to do this would be to rely on the transitional probabilities themselves, and posit a boundary whenever they are lower than a certain value. However, this would not allow us to capture dips that are relative rather than absolute. For example, while a transitional probability of 0.4 may not be extremely low, a drop in a given sequence from a transitional probability of 0.99 to one of 0.4 would indicate a meaningful dip. To capture such relative dips, we use the ratio between consecutive transitional probabilities as our cue for segmentation. That is, we calculate the ratios between each transitional probability and the previous one in a sequence, and infer a boundary when that ratio is unusually low (in other words, when the transitional probability drops dramatically). Note that ratios under 1 indicate a drop in transitional probability while ratios over 1 indicate an increase in transitional probability (see Fig. 3 for a worked example of this procedure).

We now need a way of judging when a transitional probability ratio is unusually low as to warrant cutting the sequence at that point. If we make the critical threshold for cutting too low, we risk missing real points of segmentation. If we make it too high then we risk cutting sequences that should be treated as wholes. We approach this by using the initial random sequences as a baseline: recall that this set of sequences was created to not have sequential structure (that is, the transitions between colours within a sequence are independent and not predictive). We can use this non-structured set to estimate how likely a particular drop in transitional probability is if there was no structure in the set of sequences. In this way, we can look for unexpectedly large dips in transitional probability in our data and use this as our cutting ratio. To do this, we calculate the distribution of estimated transitional probability ratios for each of the six random initial sets (one from each chain). We then create an aggregated distribution of all those ratios, and take the 5% lower tail of this ratio distribution as our cutting threshold: we cut when the ratio is lower than that (ratio = 0.425).

**Figure 4:**
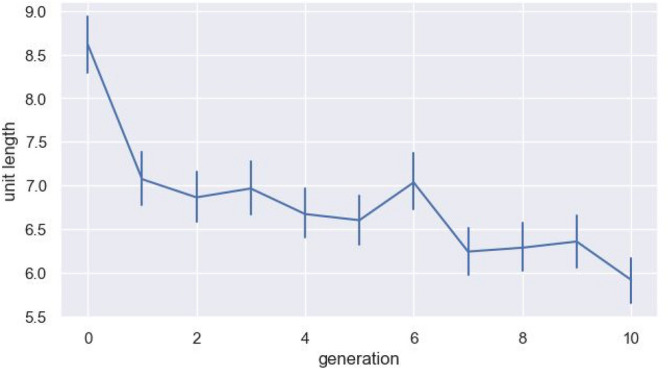
Change in the length of the units (i.e., the parts segmented by our procedure) over generations. The vertical coordinates represent the average length of all the units in a particular generation, averaged across all of the chains. Any identical units are counted multiple times. Error bars are bootstrapped 95% CIs. Units are getting shorter, indicating that smaller parts are emerging out of the larger wholes over generations.

Using this pipeline we can build an inventory of parts—a lexicon essentially—for each generation. Figure 4 shows that over generations we get a decrease in the length of the distinct units found by this method, showing that the sequences are being cut into smaller and smaller parts. In other words, the segmented units are becoming shorter. We fit a linear mixed effects model predicting unit length, with fixed effect for generation, a random intercept for chain, and random by-chain slopes for generation. We show a significant reduction in unit length over generations (β = − 0.17, sd = 0.044, *p* = 0.012). This is the case even if we remove the initial random language from the analysis (β = − 0.11, sd = 0.040, *p* = 0.041).

**Figure 5:**
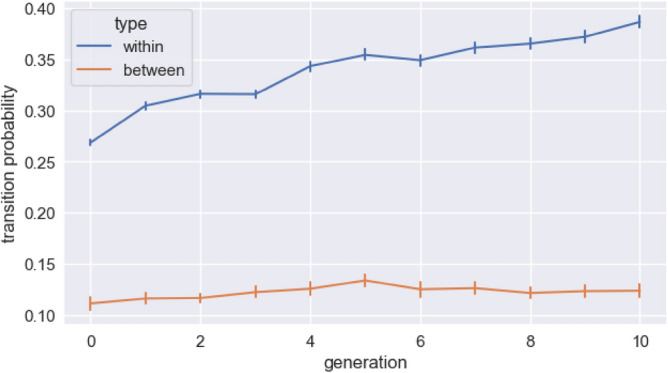
Change in the transitional probabilities within and between units over generations. Error bars are bootstrapped 95% CIs. After applying our segmentation method, we can classify each transitional probability within a sequence as being within a unit or between two units. Each point on the graph shows the means (for a particular generation across all chains) of the within-unit and between-unit transitional probabilities. Necessarily, due to the way we infer cutting points, the across-unit probabilities will be lower, even in generation zero. The key point here is that the transitional probabilities within units increase.

Importantly, we find that the within-unit transitional probabilities increase over generations, whilst the between-unit transitional probabilities remain very low, meaning that the set of sequences are evolving culturally to have increasingly clear cues to segmentation (Fig. 5). We calculate these two measures in the following way: After segmenting the sequences in each set using our pipeline, we can categorise the transitional probabilities into ones that occur within units and ones that occur between units. For example, if we had segmented the sequence “RYGR” into “RYG | R”, then the transitional probability of “G” given “RY” would be within a unit while the transitional probability of “R” given “YG” would be between units. Since we segment our sequences only when there is a dip in transitional probability, the between-unit transitions are necessarily low, however the transitional probabilities within units are increasing relative to the between-unit transitions, indicating the increased statistical coherence of the segmented units.

We fit a linear mixed effects model predicting transitional probabilities with fixed effects for generation, and type of transitional probability (within unit or between units), and their interaction, a random intercept for chain, and random by-chain slopes for generation. This shows a significant interaction of transitional probability type and generation, demonstrating that the increase in probability over generations is greater for the within-unit transitional probabilities (β = 0.010, sd = 0.00070, *p* < 0.001).

These results demonstrate that purely by transmitting sequences from one generation to the next, and despite the task not being framed as one of learning a structured set, system-wide statistical structure emerges. The system develops clearer cues to segmentation, reflected in an increasing difference between within-unit and between-unit transitional probabilities. In other words, parts are emerging from wholes.

### Statistical structure emerges

**Figure 6:**
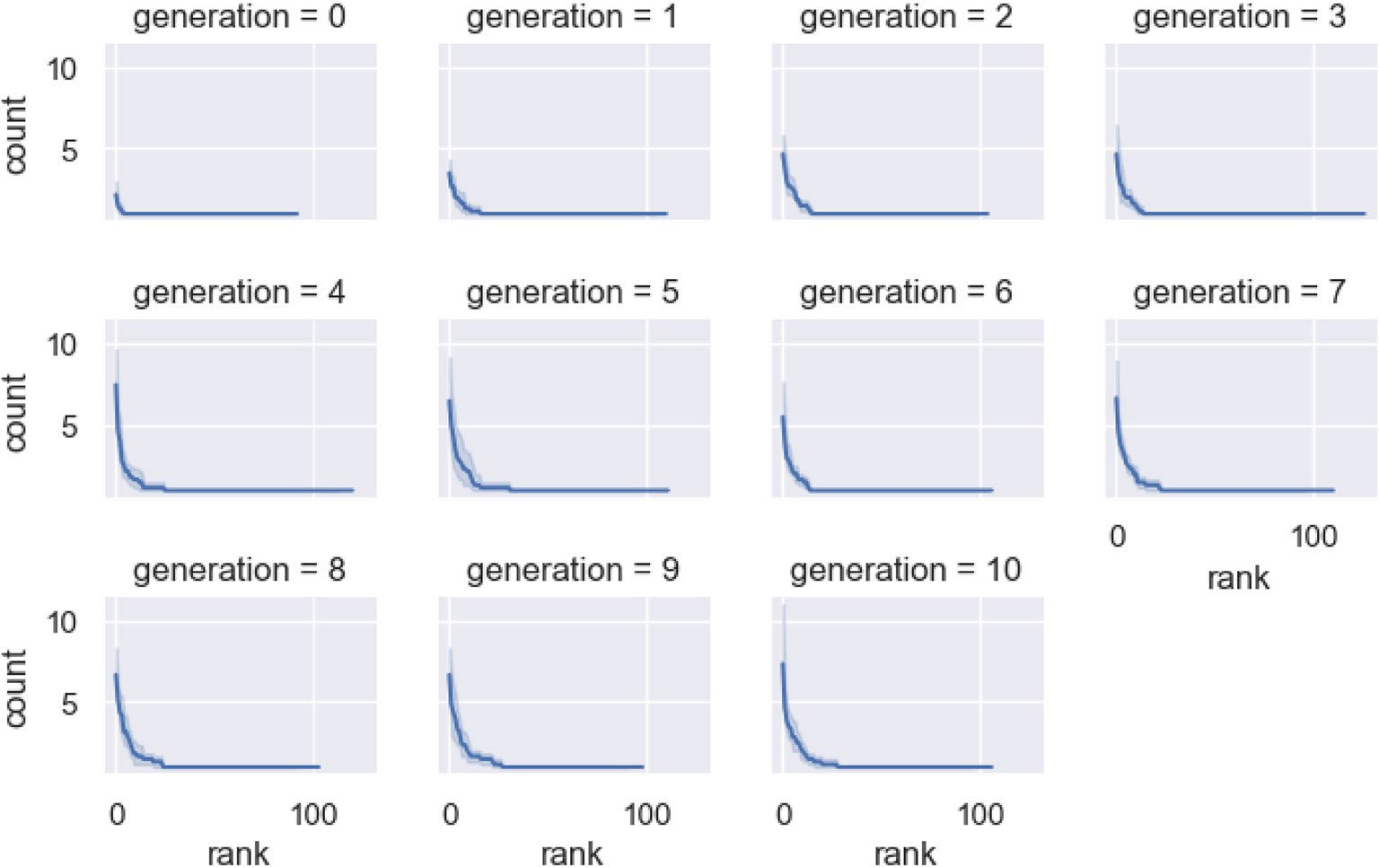
Distributions of units in the sequence sets over generations. Each is plotted as a count of how often each unit occurs against its frequency rank. Bootstrapped 95% CIs across chains are shown as a shaded region around the mean. From the initial relatively uniform distribution, a skewed distribution emerges with a small number of high frequency units and a long tail of low frequency ones.

Now that we have an inventory of units for each generation and chain in our experiment, we can ask how the frequency distribution of these units changes as the sets of sequences are transmitted by iterated learning. We do this by counting the frequency of each proposed unit in the inventory for each generation in each chain and plot that frequency against their rank in Fig. 6. This shows that from a relatively uniform starting point (with almost every item appearing only once in the random initial set), a highly skewed distribution emerges, with the most frequent units appearing between 5 and 10 times more often than the least frequent items. In other words, a skewed distribution has emerged purely as a result of transmitting these sequences from one generation to the next through iterated learning.

**Figure 7:**
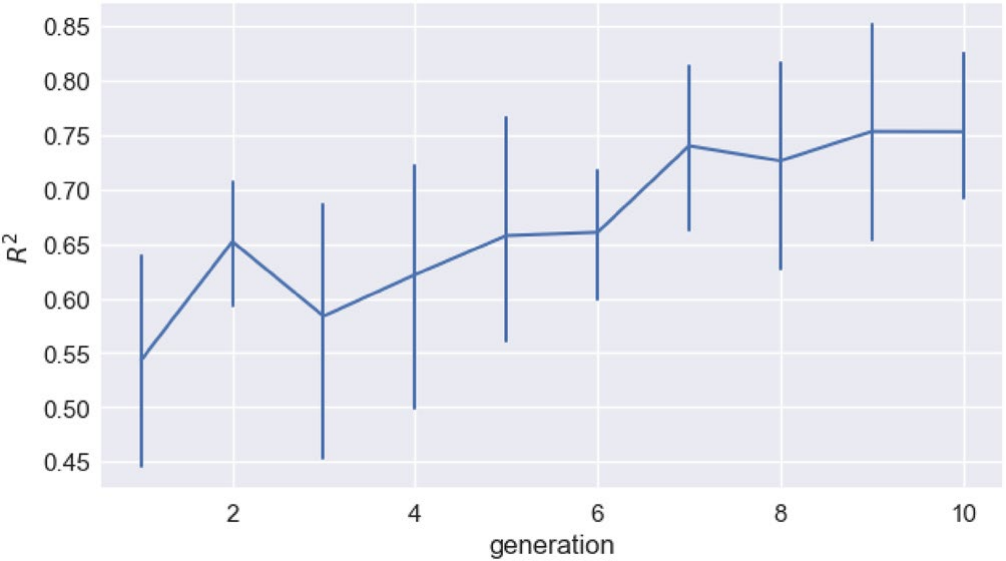
The R^2^ of the correlation between log frequency and log rank for the unit distributions given in Fig. 6, plotted against generation (generation 0, the random initial language, is removed as for many of the chains the distribution was completely flat and a correlation coefficient could not be calculated). Error bars are bootstrapped 95% CIs. Over time, word distributions are becoming more Zipfian, reflected in an increasingly linear relationship between log frequency and log rank.

We can ask how close this distribution is to a Zipfian one, and how it changes over generations, by looking at the R^2^ of log(frequency) and log(rank). For each generation in each chain, we calculate this R^2^. We can see that the fit to a Zipfian distribution increases over generations (Fig. 7). We fit a linear mixed-effects model predicting R2 with a fixed effect for generation, a random intercept for chain, and random by-chain slopes for generation. We find a significant increase in R^2^ over generations (β = 0.022, sd = 0.0066, *p* = 0.020).

**Figure 8:**
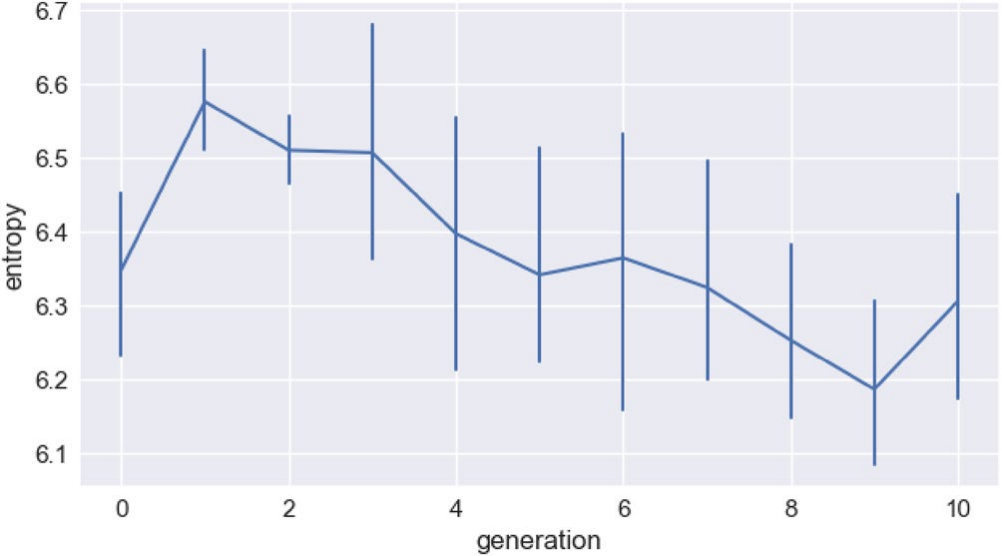
Entropy of units plotted against generation. Entropy is calculated for each generation in each chain and the average across chains is plotted. Error bars are bootstrapped 95% CIs. There is an initial increase in entropy due to an increase in inventory size (since the initial random set of sequences has very few segmentable units), but then the increasingly skewed distribution leads to a reduction in the entropy of units over generations.

We can better quantify the skew in these results by calculating the entropy of the units in the sequence sets. This gives us a simple and direct measure of how skewed the frequency distributions are. For a given number of units, a uniform distribution will have a higher entropy than a skewed distribution. Figure 8 shows that entropy decreases over generations, even when we control for the number of distinct units that are inferred by our segmentation procedure. We fit a linear mixed effects model predicting entropy, with fixed effects for generation and the number of distinct units, and a random intercept for chain (including by-chain slopes leads to a singular fit, so we use the simpler random effect structure here). We included the number of units to ensure that the effect of generation is not driven by a change in the number of units (fewer units would lead to lower entropy). We find a significant reduction of entropy by generation (β = − 0.033, sd = 0.0055, *p* < 0.0001). We also, as expected, find that entropy increases when the number of units grows (β = 0.0028, sd = 0.00084, *p* = 0.0013).

### Learnability as both cause and effect

Taken together, these results demonstrate that iterated learning can produce two consistent properties of language, the emergence of parts and their skewed distribution.

**Figure 9:**
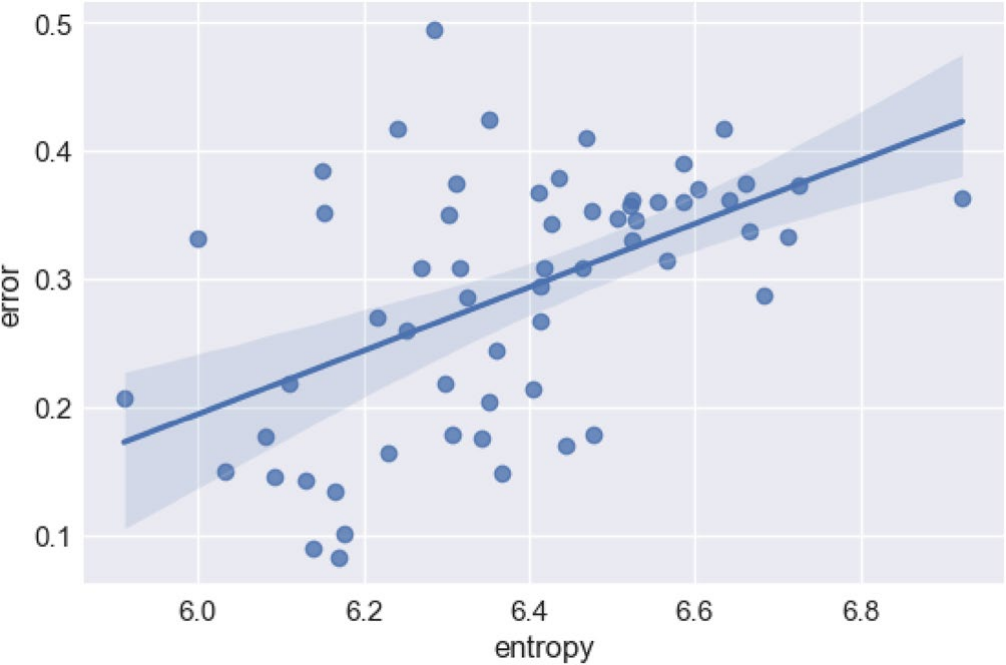
How error is related to entropy in the experiment. Each point is a single participant. The best-fit line plus 95% CIs on the fit is shown. More skewed distributions of units (i.e., sequence sets with lower entropy) are harder to learn (i.e., are copied with greater error).

We have shown that these properties can arise out of a system being transmitted by iterated learning, but can we also show that this emergent statistical structure actually improves the learnability of the system? Fig. 9 shows the relationship between the entropy of the words in the set the participant was exposed to and the error rate for that participant. There is a clear relationship between statistical structure and learnability. A Pearson’s product-moment correlation shows that error and entropy are positively correlated (r = 0.52, *p* < 0.001).

The statistical structure that emerges through iterated learning makes the systems that emerge more learnable. To put it another way, the emergence of parts and their skewed distributions are both the cause of increased learnability, and the effect of that learning iterated over generations.

## Discussion

The expressive power of language—one of our species’ most extraordinary traits—is enabled through the recombination of smaller parts into larger wholes. Unlike in many communication systems in nature, these recombinable parts of language are not innately given, but are learned by children from the language they hear. To discover the parts, children must somehow extract them from a continuous speech stream. These parts are not uniformly distributed but follow a characteristic power law distribution with a small number of high frequency words, a large number of low frequency ones, and a non-linear decrease in frequency.

In this paper we have shown that both of these properties of language—having parts and having them distributed in a particular way—can emerge through cultural transmission when learners are simply reproducing wholes. Using a non-linguistic task, we show that sets of colour sequences become more structured and more learnable over time. Specifically, the increase in structure and learnability comes from the emergence of parts whose distribution becomes more Zipfian over time. We identified these parts using transitional probabilities between colours—the same statistical measure infants use to detect word boundaries. Our findings show that the transitional probabilities within ‘units’ increases over generations, that the entropy of their distribution decreases and becomes more Zipfian over time, and that the entropy over these units is correlated with error. We propose that language gains statistically discoverable parts with an increasingly skewed distribution precisely because it is learned by multiple generations of language users. The statistical structure of language is both cause and effect of greater learnability of language.

How does this happen in our study? Our results cannot speak directly to this question, but we offer one possibility here. In our task, participants copy sequences which initially have a largely uniform statistical structure: each colour is approximately equally likely to follow any other one. While copying these patterns, participants nevertheless tend to produce some pairs of colours more frequently than others, at first purely through errors. This means that for the next generation of learners the sequences are no longer as statistically uniform. This leads to patterns of transitional probabilities where a low probability transition will sometimes follow a high probability one. Learners take this as a cue for a boundary between sub-parts of the sequence. In this way, a repertoire of unit-candidates emerges. The frequency of these unit-candidates may increase over time, creating larger differences in the probability of transitions between units and within units. This frequency advantage will be amplified from one generation to the next, leading to a more skewed distribution that is facilitative of further learning. In this ‘rich-gets-richer’ scenario, a slightly skewed distribution leads to increased utility of transitional probabilities as a cue to unit boundary, which leads to an increased repetition of the units, a more skewed distribution, and less error for the more frequent units. This process is repeated and amplified over time, leading to increased skew and a correlation between skew and learning. In this view, even though learners are faced with a task explicitly framed as one in which whole sequences are copied (with no expectations that these sequences have sub parts, let alone ones that persist over different sequences), parts are eventually created. These are recombined to create a set of sequences that are optimised for transmission over generations.

On the face of it, this whole-to-part strategy may seem counterintuitive, compared to one in which individual parts are learned first and then recombined. One reason for the existence of such a strategy is the environment itself. Parts do not come ready segmented. Language provides us with multiple such examples: phonemes can be discovered from whole-word representations^45^, morphemes from multi-morphemic words^46^, and words from multiword units^47^. We propose that a second reason for its existence is that the whole-to-part strategy can actually facilitate various aspects of learning. In language, whole-to-part can facilitate mastery of the often-arbitrary relations that hold between the parts. For example, article-noun agreement is learned better when learners start with article-noun chunks that are then segmented, than when the article and noun are learned separately and then combined^38,39^. Whole-to-part learning could also benefit other aspects of development. Infants’ initial poor visual acuity, which prevents them from perceiving visual details, has been proposed to facilitate broader spatial analysis beneficial for face processing^48^.

Note that we deliberately designed our experiment to be a non-linguistic task. The memory game is one that appears unrelated to language. The sequences have no similarity to language, and the task was not framed as one which required participants to learn about properties of a set of behaviours. Moreover, both the whole sequences and the emerging parts do not have meaning. Nevertheless, system-wide statistical structure emerges that is strikingly similar to that found in languages. This suggests that structure in language may similarly arise via cultural evolution through a highly general process of iterated sequence learning, rather than learning biases that are specifically adapted to language. We expect to see segmentable sequences that have a skewed distribution wherever those sequences are transmitted via a similar cultural process by learners who start by learning wholes and then discover parts within them. For example, since music is just such a culturally transmitted behaviour, then we would expect to observe Zipfian distributions here as well. Indeed, there is evidence that this is the case. The distribution of various elements of music, among them pitch, melodic intervals, and harmonic consonance, shows a very good fit to a Zipfian distribution (e.g.,^49^). Song is also a culturally transmitted behaviour, and one that lends itself to whole-to-part learning since songs are often learned and understood as wholes. Here also, the distribution of melodic and rhythmic elements in song worldwide follows a power-law distribution^50^.

Looking further afield, we can ask whether there are similarly skewed distributions in the communication systems of other species. If learnability is both a cause and an effect of skewed distributions, we may expect them to appear in culturally transmitted systems, especially when those systems are made up of parts and wholes. We can think of several species whose communication systems have these properties. For example, dolphins, whales, and songbirds. Interestingly, the whistle repertoires of adult bottlenose dolphins seem to follow a power-law distribution (Ref.^51^ but see^52^), as do the syllables in the song repertoires of spectacled warblers (a species of songbird^53^). Elements of humpback whale song have also been shown to have a highly skewed distribution^54^. These findings are suggestive of a similar process of structure creation operating in species other than humans. We propose that future work should look specifically at whether the facilitative effects of skewed distributions is found in non-human animals.

Finally, our work brings together distinct research traditions in cultural evolution, statistical learning, developmental psychology, and linguistics. We have shown how these may be usefully combined to provide an experimental paradigm for understanding the origins of core properties of human language. This would not have been possible without combining techniques from different areas—for example, iterated learning from the field of cultural evolution, and analysing transitional probabilities from the field of statistical learning. We believe this demonstrates the need for interdisciplinarity in building an empirically grounded theory of the origins and evolution of language.

## Data Availability

The full dataset and code for recreating all the figures and statistical analyses in this paper is available at: https://github.com/smkirby/cultural_evolution_of_statistical_structure
